# Putative Effects of Nutritive Polyphenols on Bone Metabolism In Vivo—Evidence from Human Studies

**DOI:** 10.3390/nu11040871

**Published:** 2019-04-18

**Authors:** Katharina Austermann, Natalie Baecker, Peter Stehle, Martina Heer

**Affiliations:** 1 Department of Nutrition and Food Sciences, Nutritional Physiology, University of Bonn, 53115 Bonn, Germany; kataust@uni-bonn.de (K.A.); pstehle@uni-bonn.de (P.S.); 2 IUBH International University, 53604 Bad Honnef, Germany; n.baecker@IUBH-fernstudium.de

**Keywords:** polyphenols, antioxidants, flavonoids, bone, osteoporosis, bone loss

## Abstract

For the prevention and treatment of bone loss related diseases, focus has been put on naturally derived substances such as polyphenols. Based on human intervention studies, this review gives an overview of the effects of dietary significant polyphenols (flavonoids, hydroxycinnamic acids, and stilbenes) on bone turnover. Literature research was conducted using PubMed database and articles published between 01/01/2008 and 31/12/2018 were included (last entry: 19/02/2019). Randomized controlled trials using oral polyphenol supplementation, either of isolated polyphenols or polyphenols-rich foods with healthy subjects or study populations with bone disorders were enclosed. Twenty articles fulfilled the inclusion criteria and the average study quality (mean Jadad score: 4.5) was above the pre-defined cut-off of 3.0. Evidence from these studies does not allow an explicit conclusion regarding the effects of dietary important polyphenols on bone mineral density and bone turnover markers. Differences in study population, habitual diet, lifestyle factors, applied polyphenols, used doses, and polyphenol bioavailability complicate the comparison of study outcomes.

## 1. Introduction

The human skeleton is continuously remodeled throughout life by osteoclast- (bone resorbing cells) and osteoblast (bone forming cells) activities [1]. Bone remodeling ensures mineral homeostasis, maintains the integrity of the skeleton, and is responsible for removal and repair of damaged tissue [2]. The underlying close communication and interaction between osteoclasts and osteoblasts consist of four consecutive phases: activation, resorption, formation, and termination/resting [2,3]. In brief, during the activation phase, an initiating remodeling signal is detected by bone cell receptors supporting the migration of partially differentiated mononuclear preosteoclasts to the bone surface. Multinucleated osteoclasts are then formed promoting resorption of bone mass. In the third phase mononuclear cells prepare the bone surface for the osteoblast-mediated formation and initiate osteoblast differentiation and –migration. Osteoblasts replace the removed bone with an equal quantity of new bone. Flattened lining cells cover the surface and mineralization occurs [2,3].

The main regulators of bone turnover are mechanical strain, systemic factors (e.g., calcitriol, calcitonin, growth hormone, insulin-like growth factor 1, glucocorticoids, and sex hormones), and local factors (e.g., the osteoprotegerin [OPG] - receptor activator of nuclear factor-kappa B ligand [RANKL] - receptor activator of nuclear factor-kappa B [RANK] system) [4,5,6]. 

**Figure 1:**
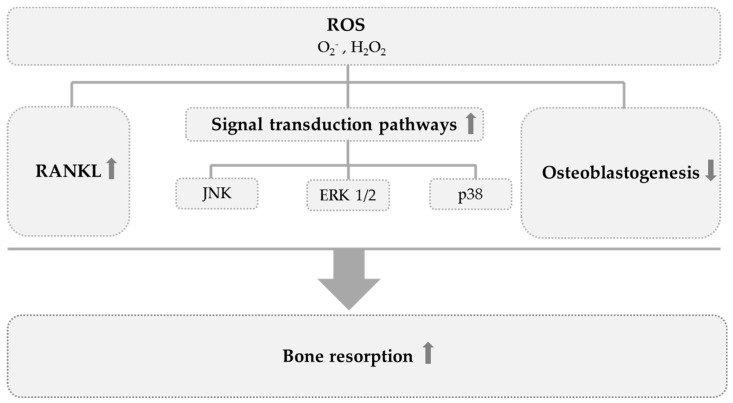
**Impact of reactive oxygen species (ROS) on bone turnover** [8,9,10,11,12,13,14]. ROS promote bone resorption by enhancing receptor activator of nuclear factor-kappa B ligand [RANKL]-induced osteoclast activity, by activation of osteoclastogenesis related signal transduction cascades (c-Jun N-terminal kinase (JNK), p38 mitogen-activated protein kinases (p38), extracellular signal-regulated kinase (ERK 1/2)), and by suppressing osteoblastogenesis. ↑, activation; ↓ inhibition.

Aside from these, the physiological balance between oxidants and antioxidants (redox status) also seems to be important for the maintenance of a balanced osteoclast- and osteoblast activity and therefore a successful bone remodeling process (Figure 1) [7,8]. Several in vitro and animal studies have shown that reactive oxygen species (ROS) production is involved in the regulation of bone status and in mineral tissue homeostasis mainly by promoting bone resorption [9,10,11,12]. Moreover, ROS act as signaling molecules in several signaling pathways in bone cells and enhance osteoclastogenesis (Figure 1) [8].

Under normal physiological conditions the ROS production by osteoclasts contributes to bone remodeling by stimulating the destruction of calcified tissue [13,14]. Exceeding ROS production and osteoclastic activity, however, were observed in different skeletal pathologies such as osteoporosis and bone fractures [15]. Several studies indicate a relation between oxidative stress and bone loss [16,17,18,19]. Oxidative stress associated with increased lipid peroxidation seems to enhance bone resorption resulting in reduced bone mineral density (BMD) [16,17]. A higher value of the superoxide dismutase (SOD)/glutathione peroxidase (GPx) ratio was observed in subjects with osteoporosis [18]. SOD generates H_2_O_2_ by removing superoxide and therefore has to collaborate with H_2_O_2_-removing enzymes like GPx or catalase to prevent oxidative stress [20]. The imbalance created by an altered SOD/GPx ratio leads to an increase in H_2_O_2_ levels [21]. High H_2_O_2_ levels promote osteoclastic differentiation and inhibit osteoblastic differentiation, which results in bone resorption [22,23].

Numerous observational studies have shown that intake of several portions of fruits and vegetables per day (~240—400 g) is associated with greater BMD and decreased fracture risk [24,25,26]. Recent reviews summarizing observational studies in Asia conclude that the consumption of soy isoflavonoids is inversely associated with the incidence of hip fractures and osteoporosis risk in postmenopausal women [27,28]. Epidemiological studies focusing on tea drinking (green- and black tea) show adverse results with respect to bone health [29,30,31,32,33]. Observational studies evaluating the effects of habitual tea drinking on bone health showed, however, inconsistent results in both men and women [34]. The generally positive effects of fruit-, vegetable-, and tea consumption seem to be partly attributed to their content of alkaline-precursors which contribute to neutralizing acid loads from other components of the diet so that the skeleton is not used as a buffer to resorb and neutralize acid loads [35]. 

More important might be their content of active phytochemical compounds, such as polyphenols [36,37,38,39]. Due to their antioxidative potential, polyphenols may protect cells against oxidative damage induced by ROS and thereby attenuate the risk for the development of degenerative diseases such as cardiovascular diseases, cancer, diabetes, and osteoporosis [40,41]. In vitro- as well as animal studies suggest that polyphenols, apart from their antioxidative properties, affect bone metabolism by anti-inflammatory actions, suppression of osteoclastogenesis, and activation of osteoblastogenesis via different bone related pathways [42,43,44,45,46,47,48,49,50]. 

**Figure 2:**
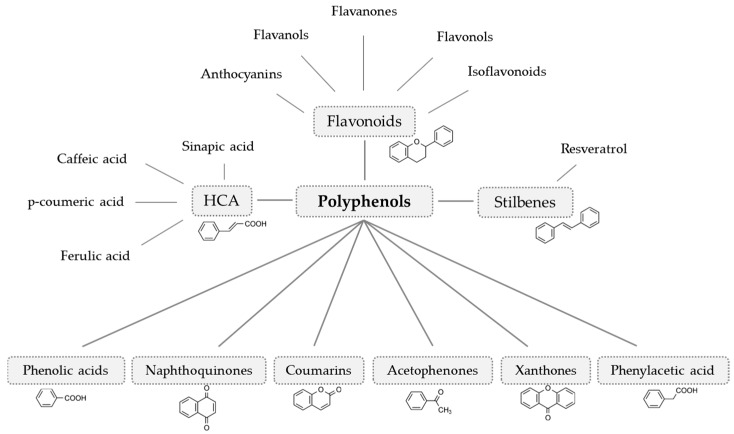
**Polyphenol classification** (modified from Crozier et al. [51]) The nine polyphenol subgroups are classified according to their chemical structure and are found throughout the plant kingdom. HCA, hydroxycinnamic acids.

Polyphenols can be distinguished according to their chemical structure (number and arrangement of carbon atoms). Based on that, they can be classified into nine subgroups (Figure 2) [51]. Depending on the amount of vegetables and fruits consumed, the daily intake of polyphenols sums up to >500 mg/day (five portions of vegetables and fruits per day). The additional consumption of tea (green-, black-, white-, and Oolong tea), coffee, and cocoa can lead to intakes up to 1000—1500 mg/day [52]. 

Relevant nutritive polyphenol subgroups are flavonoids, hydroxycinnamic acids, and stilbenes [51]. Flavonoids are found in a variety of fruits, vegetables, herbs, and beverages. [53]. The most abundant flavanols are (+)-catechin, (-)-epicatechin (EC), (+)-gallocatechin (GC), and (-)-epigallocatechin (EGC) and the gallic acid esters (-)-epicatechin gallate (ECG) and (-)-epigallocatechin gallate (EGCG). Tea (camellia sinensis) is the most quantitative source of these compounds worldwide [53]. The predominating flavonols are quercetin, kaempferol, myricetin, and isorhamnetin [53]. They usually occur as glycosides and are mainly located in the flowers, leaves, and outer parts of the plant as peel or skin. Important dietary sources are onions, apples, and leafy vegetables [54,55]. Flavanones are mainly found in citrus fruits [56]. The dominant flavanone in lemon, mandarin, and sweet orange is the rutinoside hesperidin. Sour oranges and grapefruits are dominated by the neohesperidoside naringin [57]. Major flavones are luteolin and apigenin. They are usually present as O- and C-glycosides. Aglycons of flavones are not found in fresh plants but can occur after processing [53]. Luteolin and apigenin have been identified in several vegetables such as celery and artichoke and in different herbs such as rosemary, thyme, or parsley [58,59]. Isoflavonoids, such as genistein, daidzein, and glabridin are also referred to as phytoestrogens due to their estrogenic activity. Important dietary sources for genistein and daidzein are legumes such as soybeans [60]. Glabridin is an isoflavan found in the licorice root [61]. Anthocyanins are responsible for the red, blue, or purple color of several fruits and vegetables such as plums, cherries, raspberries, blackberries, blackcurrants, beetroot, and red cabbage [62]. Aglycons, such as cyanidin or delphinidin are rarely found in plants and most commonly bounded sugars are glucose, galactose, rhamnose, and arabinose, usually as 3-glycosides [63]. Hydroxycinnamic acids (HCA) are also widely found in the human diet and main derivates are caffeic-, ferulic-, ρ-coumaric,- and sinapic acid. They usually occur as esters or glycosides of quinic acid. O-glycosylated ferulic-, caffeic-, and ρ-coumaric acids are present in tomatoes [64]. Other fruits containing hydroxycinnamic acids are plums, blueberries, cherries, and apples [65]. Stilbenes are present in vegetables and fruits such as spinach, berries, apples, and grapes. In plants, they are produced in response to stress, injury, or disease. The parent compound resveratrol can occur in *cis*- and *trans* configuration, as glucosides, aglycones, monomers, or polymers [66]. In higher concentrations resveratrol can be found in red grapes and, thus, in red wine, depending on the species [67].

The role of nutritive polyphenols in maintaining bone health is not finally resolved. Indeed, a final conclusion of the qualitative and quantitative role of nutritive polyphenols on bone metabolism and bone health can only be made on the basis of intervention studies. Thus, the aim of this literature review is to summarize and evaluate results of recently published human intervention studies investigating the effects of nutritive polyphenols, either as single substrates or as ingredients of foods, on bone metabolism.

## 2. Methods

The systematic literature search (U.S. National Library of Medicine National Institutes of Health online database PubMed) sought to identify all eligible English articles published between 2008 and 2018 in peer-review journals (last entry: 19/02/2019) with a clear focus on the major polyphenol subgroups. The following search terms were used and at least one of the terms in each of the following four lists had to be present in the title and/or abstract of the article: (1) clinical, experimental, human, in vivo, intervention; (2) bone, bone turnover, bone markers, bone loss; (3) nutrition, nutritional, supplementation, oral; (4) polyphenols, flavonoids, flavanols, flavonols, flavanones, flavones, isoflavonoids, isoflavones, anthocyanins, stilbenes, hydroxycinnamic acids. The following PubMed filters were applied: publication date (from 01/01/2008 to 31/12/2018) and species (humans). In addition, reference lists of articles identified during the literature search have been checked for complete identification of eligible articles.

### 2.1. Article Selection 

**Figure 3:**
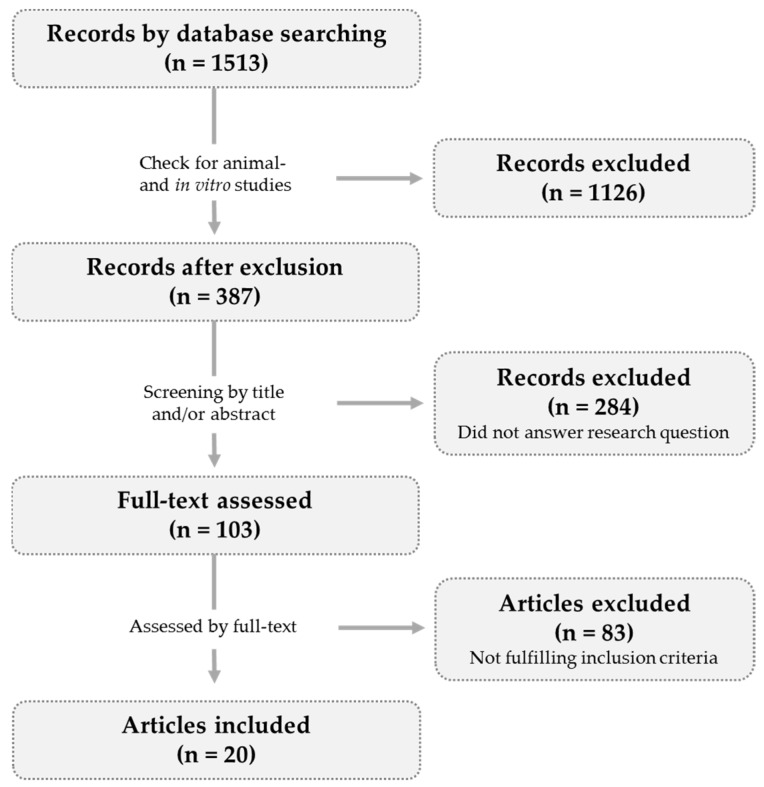
**Study selection diagram.** The literature search revealed 1513 hits (PubMed filters: publication date (from 01/01/2008 to 31/12/2018) and species (humans). After removal of further animal- and in vitro studies 387 records were screened. Full-text was assessed for 103 records and 83 articles did not meet the inclusion criteria. Twenty articles were included.

Studies meeting the following inclusion criteria were included in the evaluation: (a) randomized controlled trials; (b) oral polyphenol supplementation; (c) supplementation of isolated polyphenols or polyphenol-rich foods; (d) healthy subjects or study populations with bone loss related diseases (e) outcomes: BMD or bone turnover markers; (f) publication date: 2008—2018. As shown in Figure 3, 20 articles were finally included in this review.

Two independent and experienced reviewers manually screened the title and/or the abstract of the articles that were flagged during the literature search for adherence to the above eligibility criteria. When the reviewers disagreed about the eligibility of a particular article the whole text of the article was read and a consensus decision was reached.

### 2.2. Data Presentation

Data extraction followed a predefined protocol. Human trials were categorized according to the polyphenol subclass (flavanols, flavonols, flavanones, flavones, isoflavonoids, anthocyanins, hydroxycinnamic acids, stilbenes) administered, or in the case of food consumption, according to the dominant polyphenol ingredient of the food items under investigation. To evaluate study quality the Jadad score was calculated for each study included [68]. In this score randomization, blinding, and dropout description are assessed. The scale ranges from 0 (low quality) to 5.0 (high quality) [68]. Scores above a defined cut-off of 3.0 indicate that reliable conclusions can be drawn.

## 3. Results and Discussion

Study details of the included studies are summarized in Table 1. The volunteer characteristics, intervention protocols, characterization of the control group, study duration, and observed effects on bone are shown. Most of the human trials were performed in postmenopausal women and participant numbers range from twelve to 431. Time of intervention varied between eight weeks and three years and health status of volunteer collectives differed.

The number of intervention studies conducted for the different polyphenol subgroups differ broadly (one study each for flavanols and stilbenes and ten studies for isoflavonoids). The main class of flavonoids investigated for their potential effects on bone metabolism is isoflavonoids because of their structural similarity to estrogen and their ability to bind to the estrogen receptor [89]. Another reason for the higher number of studies for this polyphenol subgroup might be their dietary significance particularly in Asian countries and for vegetarian- and vegan lifestyles.

The sample size of the included studies vary between twelve volunteers [72] and 431 subjects [73] and study durations range from two months [71] to two years [75]. Most studies (except four) conducted a power calculation prior to the beginning of the study. 

Apart from that, outcome variables investigated differ broadly. Most studies examined BMD of volunteers [69,71,73,78], whereas other investigators analyzed different markers of bone turnover [70,72,80]. Bone turnover markers, such as bone alkaline phosphatase (bAP), aminoterminal propeptide of type I collagen (P1NP), C-telopeptide of type I collagen (CTX), and N-telopeptide of type I collagen (NTX) are beside the BMD good indicators for fracture risk. They are sometimes even stronger associated with this risk than BMD, as they predict fractures in two different ways: (1) the direct reduction of BMD via high bone turnover and (2) independently of BMD, by affecting bone microarchitecture and -fragility [90]. Bone markers are also often used to monitor anti-resorptive therapies and provide a good method for the investigation of nutritional interventions, as changes can be observed more rapidly compared to BMD [90]. As summarized by Eastell et al. early changes in bone turnover markers may be predictive of BMD changes [90]. Reduction of CTX- and NTX concentrations, for instance after six months predict an increase in lumbar spine BMD 2.5-4 years later and an increase of P1NP after three months is associated with changes in lumbar spine BMD after 18 months [90]. For shorter intervention periods (two to three months) it, therefore, might be reasonable to accompany the investigation of BMD with the examination of bone turnover markers as BMD changes might not be observed at this time point. Six of the nine studies that investigated the effects of BMD and bone turnover markers observed similar effects on these parameters (e.g., no changes for both outcomes) [75,76,77,78,81,86,87,88]. Three studies investigating both outcomes showed contradictory results [71,83,84,85]. Law et al. did not find any changes in total body BMD but observed a reduction in the bone formation marker bAP after consumption of 100 ml onion juice per day for two months [71]. The study duration might not be long enough to already see changes in BMD. Hooshmand et al. found an increase of ulna- and spine BMD after one year of dried plum consumption [84,85]. The changes for OPG and sclerostin they observed were not statistically significant but showed a trend in the same direction [84,85].

Studies examining the effects on bone metabolism in healthy volunteers (prevention of bone loss) did not find any beneficial effects [71,72,73,78,79,82]. Only one study investigating the effects in healthy women found a smaller reduction in whole-body BMD after 2 years of soy isoflavonoid supplementation (120 mg/d) compared to placebo [83]. However, the authors stated that the difference only translates to a minimal clinical effect and the supplementation did neither slow bone loss at key fracture sites nor affected bone marker concentrations [83]. Studies that investigated the effect of polyphenols as a treatment for osteopenic women (therapeutic effect) observed a positive impact on bone metabolism [70,75,84,86]. One might speculate that polyphenols may only have a therapeutic- but no preventive effect. However, further studies are needed to investigate and confirm this observation.

Doses applied show a high variation between the different studies (several mg up to 1 g per day). Results, however, do not indicate a dose-dependent effect, as 843 mg EGCG did not affect BMD [69], whereas 54 mg genistein improved BMD [75,76]. It has to be taken into account that we here compare different polyphenol subgroups. They might have a different potency and therefore different doses are needed.

Variations in study population (ethical background and age of participants), habitual diet (substituted polyphenols might not have an additional effect if volunteers already have a balanced diet), and lifestyle factors such as physical activity are other factors that might impact study results and lead to contrary findings.

A comparison between human- and animal studies shows that human intervention studies did not consistently confirm the beneficial effects found in animal models. The transferability of results from animal models to humans, however, is limited, because of differences in e.g., physiology, metabolism and bioavailability. It is likely that animals and humans metabolize polyphenols differently. This has to be considered in the evaluation of these results. Moreover, supra-nutritional doses are mostly used in animal studies and these amounts are not attainable within a plant-based diet by humans.

The bioavailability might be a further explanation of inconsistent study results. Bioavailability of polyphenols depends on external (e.g., food related factors and chemical structure) and internal factors (gender, age, colonic microflora, etc.) [91]. Interactions with other food components, such as fat, proteins, or other polyphenols, for instance, can affect the bioavailability of a single compound [92]. This is important for the valuation, particularly, of those studies investigating the effects of a single compound on bone metabolism. The presence of other polyphenols for example seems to increase the polyphenol bioavailability [92]. Therefore, it might be interesting to investigate whether the effective dose of single compounds can be reduced if they are applied with other polyphenols or as polyphenols-rich foods.

## 4. Conclusions

Obviously, recent intervention studies investigating the effects of nutritive polyphenols, either ingested via food or given as single compounds, on bone health showed inconsistent results. Consequently, final conclusions cannot be drawn. Differences in study population, habitual diet, lifestyle factors, and polyphenol bioavailability complicate the comparison of study outcomes. Future studies should take these confounding factors into account. Moreover, it might be of specific interest to evaluate whether the application of polyphenol mixtures (supplements) can lead to beneficial synergistic effects.

## Figures and Tables

**Table 1:** Overview of human intervention studies included.

	Participants				Intervention	Control Group	Duration	Power Analysis	Effects on Bone	Jadad Score
	Number	Age (Year)	Gender	Health Status	(Powder/Food Item)					
Flavanols										
Dostal et al. 2016 [69]	121	50–70	Female	Overweight/obese, postmenopausal, high breast cancer risk	GTE (843 mg EGCG/d)	Overweight/obese, postmenopausal women with high breast cancer risk	1 year	Yes (80%)	Total body BMD ↔	5
Shen et al. 2012 [70]	171	>50	Female	Postmenopausal, osteopenic	GTE (500 mg/d)	Postmenopausal, osteopenic women	6 months	Yes (85-90%)	bAP ↑ TRAP ↔ bAP/TRAP ratio ↑	5
Flavonols										
Law et al. 2016 [71]	30	40–80	Female, male	Healthy	Onion juice (100 ml/d)	Healthy men and women	8 weeks	No	Total body BMD ↔ bAP ↓ PTH ↔ Calcium ↔	5
Flavanones										
Martin et al. 2016 [72]	12	>50	Female	Postmenopausal, healthy	Hesperidin (500 mg)	Postmenopausal, healthy women	3 months	Yes (80%)	bAP ↔ DPD ↔	5
Isoflavonoids										
Alekel et al. 2010; Shedd-Wise et al. 2011 [73,74]	255	46–65	Female	Postmenopausal, healthy	Soy isoflavonoids (80 and 120 mg/d)	Postmenopausal, healthy women	3 years	Yes (94%)	Total body BMD ↔ spine BMD ↔ femur BMD ↔ neck BMD ↔	5
Arcoraci et al. 2017; Marini et al. 2008 [75,76,77]	389	49–67	Female	Postmenopausal, osteopenic	Genistein (54 mg/d)	Postmenopausal, osteopenic women	2 years	Yes (80%)	Femur BMD ↑ spine BMD ↑ PYD ↓ DPD ↓ bAP ↑ RANKL ↓ OPG ↑	5
Brink et al. 2008 [78]	237	53±3	Female	Early postmenopausal, healthy	Isoflavonoid enriched foods (110 mg isoflavonoid aglycones/d)	Early postmenopausal, healthy women	1 year	Yes (84%)	Total body BMD ↔ bone markers ↔	5
Kenny et al. 2009 [79]	131	>60	Female	Postmenopausal, healthy	Isoflavonoids (105 mg/d)	Postmenopausal, healthy women	1 year	No	Total body BMD ↔ femur BMD ↔ spine BMD ↔ wrist BMD ↔	4
Sathyapalan et al. 2016 [80]	200	>50	Female	Early postmenopausal	Isoflavonoids (66 mg/d)	Early postmenopausal women	6 months	Yes (95%)	ßCTX ↓ P1NP ↓	5
Tai et al. 2012 [81]	431	45–65	Female	Postmenopausal with bone loss	Isoflavonoids (300 mg/d)	Postmenopausal women with bone loss	2 years	Yes (80%)	Femur BMD ↔ Bone markers ↔	5
Vupadhyayula et al. 2009 [82]	203	>50	Female	Postmenopausal, healthy	Isoflavonoids (90 mg/d)	Postmenopausal, healthy women	2 years	Yes (80%)	Spine BMD ↔ Femur BMD ↔	4
Wong et al. 2009 [83]	403	40–60	Female	Climacteric, healthy	Soy isoflavonoids (80 and 120 mg/d)	Climacteric, healthy women	2 years	Yes (80%)	Total Body BMD ↑ (120 mg/d) Bone markers ↔	5
Anthocyanins										
Hooshmand et al. 2011 and 2014 [84,85]	160	>50	Female	Postmenopausal, osteopenic	Dried plums (100 g/d)	Postmenopausal, osteopenic women	1 year	No	Ulna BMD ↑ Spine BMD ↑ OPG ↔ Sclerostin ↔	3
Hooshmand et al. 2016 [86]	48	65–79	Female	Postmenopausal, osteopenic	Dried plums (50 and 100 g/d)	Postmenopausal, osteopenic women	6 months	No	Total BMD ↑ TRAP ↓	3
Simonavice et al. 2014 [87]	27	64 ± 7	Female	Postmenpausal, breast cancer survivors	Dried plums (90 g/d)	Postmenpausal women, breast cancer survivors	6 months	Yes (80%)	Spine BMD ↔ Femur BMD ↔ Forearm BMD ↔ Bone markers ↔	3
Stilbenes										
Ornstrup et al. 2014 [88]	74	49 ± 6	Male	Obese, metabolic syndrome	Resveratrol (150 and 1000 mg/d)	Obese men with metabolic syndrome	16 weeks	Yes (80%)	Spine BMD ↑ (1000 mg/d) bAP ↑ (1000 mg/d) OPG ↔ P1NP ↔ CTX ↔ NTX ↔	5

↔, no changes; ↑, significant increase; ↓, significant reduction; GTE, green tea extract; BMD, bone mineral density; bAP, bone alkaline phosphatase; TRAP, tartrate-resistant acid phosphatase; PTH, parathyroid hormone; DPD, desoxypyridinolin; PYD, pyridinolin; RANKL, receptor activator of nuclear factor-kappa B ligand; ßCTX, ß C-telopeptide of type I collagen; P1NP, aminoterminal propeptide of type I collagen; OPG, osteoprotegerin; CTX, C-telopeptide of type I collagen; NTX, N-telopeptide of type I collagen; d: day.
